# The role of lung ultrasound B-lines and serum KL-6 in the screening and follow-up of rheumatoid arthritis patients for an identification of interstitial lung disease: review of the literature, proposal for a preliminary algorithm, and clinical application to cases

**DOI:** 10.1186/s13075-021-02586-9

**Published:** 2021-08-14

**Authors:** Yukai Wang, Shaoqi Chen, Shaoyu Zheng, Jianqun Lin, Shijian Hu, Jinghua Zhuang, Qisheng Lin, Xuezhen Xie, Kedi Zheng, Weijin Zhang, Guangzhou Du, Guohong Zhang, Anna-Maria Hoffmann-Vold, Marco Matucci-Cerinic, Daniel E. Furst

**Affiliations:** 1 Department of Rheumatology and Immunology, Shantou Central Hospital, Shantou, Guangdong China; 2 Department of Experimental and Clinical Medicine & Division of Rheumatology, Azienda Ospedaliero Universitaria Careggi, University of Florence, Florence, Italy; 3 Department of Geriatric Medicine, Division of Rheumatology, AOUC, Florence, Italy; 4 Department of Ultrasound, The First Affiliated Hospital of Shantou University Medical College, Shantou, Guangdong China; 5 Department of Radiology, Shantou Central Hospital, Shantou, Guangdong China; 6 Department of Pathology, Shantou University Medical College, Shantou, Guangdong China; 7 Department of Rheumatology, Oslo University Hospital, Oslo, Norway; 8 Division of Rheumatology, Department of Medicine, University of California at Los Angeles, Los Angeles, USA

**Keywords:** Lung ultrasound, B-lines, KL-6, High-resolution computed tomography, Pulmonary function tests, Rheumatoid arthritis–associated interstitial lung disease, Screen, Follow up

## Abstract

Screening and follow-up of interstitial lung disease associated with rheumatoid arthritis (RA-ILD) is a challenge in clinical practice. In fact, the majority of RA-ILD patients are asymptomatic and optimal tools for early screening and regular follow-up are lacking. Furthermore, some patients may remain oligosymptomatic despite significant radiological abnormalities. In RA-ILD, usual interstitial pneumonia (UIP) is the most frequent radiological and pathological pattern, associated with a poor prognosis and a high risk to develop acute exacerbations and infections. If RA-ILD can be identified early, there may be an opportunity for an early treatment and close follow-up that might delay ILD progression and improve the long-term outcome.

In connective tissue disease–associated interstitial lung disease (CTD-ILD), lung ultrasound (LUS) with the assessment of B-lines and serum Krebs von den Lungen-6 antigen (KL-6) has been recognized as sensitive biomarkers for the early detection of ILD. B-line number and serum KL-6 level were found to correlate with high-resolution computed tomography (HRCT), pulmonary function tests (PFTs), and other clinical parameters in systemic sclerosis–associated ILD (SSc-ILD). Recently, the significant correlation between B-lines and KL-6, two non-ionizing and non-invasive biomarkers, was demonstrated. Hence, the combined use of LUS and KL-6 to screen and follow up ILD in RA patients might be useful in clinical practice in addition to existing tools. Herein, we review relevant literature to support this concept, propose a preliminary screening algorithm, and present 2 cases where the algorithm was used.

## Introduction

Rheumatoid arthritis (RA) is a systemic, autoimmune, and inflammatory joint disease characterized by synovitis and bone erosion [1]. The lung is the major site of extra-articular involvement. Though RA can affect all compartments of the respiratory system, its effects on the parenchyma, especially interstitial lung disease (ILD), can result in significant morbidity and mortality [2]. The most common radiological and histopathologic pattern of RA-ILD is usual interstitial pneumonia (UIP), characterized radiographically by honeycombing, reticulation, and traction bronchiectasis, with basilar and peripheral predominance [3, 4]. Similar to idiopathic pulmonary fibrosis (IPF) [5], the clinical behavior of RA-related UIP is highly variable, associated with a high risk of acute exacerbations and a notoriously poor response to treatment [6–8]. In addition, the radiologic pattern of UIP in RA-ILD predicts poorer prognosis and higher mortality compared to the non-specific interstitial pneumonia (NSIP) pattern, which is most commonly seen in other connective tissue disease–related ILD (CTD-ILD) [9–11]. Notably, in certain clinical scenarios, respiratory symptoms do not necessarily parallel radiographic findings and/or pulmonary function tests (PFTs). Some patients remain asymptomatic or oligosymptomatic despite significant chest radiographic abnormalities and impaired lung function.

Accordingly, early screening of RA patients could help to identify early stages of RA-ILD. However, the present assessment tools including chest X-ray, high-resolution computed tomography (HRCT), and PFTs may not be optimal tools for screening purposes [12, 13]. In the past decade, lung ultrasound (LUS) B-lines and serum Krebs von den Lungen-6 antigen (KL-6) have been recognized as possible novel sensitive biomarkers to detect CTD-ILD [14–16]. In addition, a significant correlation between B-line number and serum KL-6 level was demonstrated recently in patients with CTD-ILD and idiopathic inflammatory myositis–related ILD (IIM-ILD) [17, 18]. Based on this background, we believe it might be helpful to integrate LUS and KL-6 to screen and follow up RA patients with ILD.

Here, we review the relevant literature on measures to define RA-ILD to present some evidence supporting this approach. Thereafter, we propose a preliminary algorithm to screen and follow up early RA-ILD, combining LUS, KL-6, HRCT, PFTs, and clinical signs and symptoms. Next, we present two cases in which LUS and KL-6 effectively identified early asymptomatic ILD. Finally, we suggest a study to examine LUS and KL-6 to define their usefulness and validation in clinical application.

## Review of relevant medical literature

### Unmet needs in RA-ILD

ILD, a severe fibrotic disease of the lung parenchyma, is one of the most common causes of death in RA patients [19]. However, our knowledge regarding the precise prevalence, pathogenesis, and natural history of RA-ILD is poorly understood. Heterogeneous disease course and the lack of optimal screening tools and guidelines make early diagnosis and proper intervention challenging. Early RA-ILD patients are frequently asymptomatic [20, 21]. When cough and dyspnea appear (associated with ILD and not due to other causes), the disease may already be advanced and is associated with a poor prognosis [11]. In addition, the current mainstay treatment regimens of RA such as methotrexate, leflunomide, and anti-tumor necrosis factor-α (TNF-α) agents might play a possible role in lung injury and exacerbation of existing ILD [22–24]. However, the use of methotrexate in RA-ILD is controversial and has recently also been associated with increased survival and a decreased risk of ILD in RA [25, 26]. Finally, to our knowledge, there are few ongoing and completed randomized, double-blind, placebo-controlled therapeutic trials including patients with RA-ILD [27, 28], and no treatment recommendations exist. Treatment approaches in clinical practice for RA-ILD are largely based on data derived from SSc-ILD or IIM-ILD, individual physician’s experience, and published case reports and series [21, 29].

### Genetic, environmental, and demographic risk factors for RA-ILD

In the past decade, studies found that genetic variants have been implicated in the development of RA-ILD. A gain-of-function promoter variant (rs35705950) in the mucin 5B (MUC5B) gene was associated with RA-ILD, more specifically associated with evidence of UIP [30]. HLA-B54, HLA-DQ1B*0601, HLA-B40, and the site encoding α-1 protease inhibitor are associated with an increased risk of RA-related ILD [31]. An excess of mutations was observed in telomere maintenance genes (TERT, PARN, RTEL1) and in SFTPC, involved in surfactant homeostasis, with increased odds ratios (OR) of 3.17 (95% CI 1.53–6.12; *p* = 9.45 × 10^−4^) for ILD as compared with controls [32].

Published data, mostly from retrospective studies, has identified several environmental and demographic risk factors that predict RA-ILD development, including male sex, older age, older age at RA diagnosis, tobacco smoking, high disease activity, seropositivity and titer of rheumatoid factor (RF) and/or anti-cyclic citrullinated peptide (CCP) antibodies, and the presence of other non-pulmonary extra-articular manifestations [20, 33, 34]. Other RA-ILD-associated antibodies have also been described to be associated with ILD, including anti-carbamylated proteins and other post-translational modified proteins [35].

### Conventional diagnostic tools for CTD-ILD

Because respiratory symptoms occur often late and are frequently unspecific, it is not advised to screen patients for ILD based on symptomatology. Usually, a chest X-ray is the initial examination performed to evaluate suspected lung involvement. It is frequently performed in RA patients prior to initiating methotrexate and biologic agent treatment. X-ray has a good specificity for ILD diagnosis, but a very low sensitivity limiting its role for ILD screening purposes [36, 37].

HRCT is the gold standard for ILD diagnosis and evaluation of disease severity of ILD. HRCT can identify even subtle ILD changes. Serial HRCT scans can be performed to monitor existing diseases. However, radiation exposure and high cost are two limiting factors in the use of HRCT [38, 39], especially for screening purposes in younger patients and for monitoring over time. Recently, to reduce radiation dose, a newly proposed 9-slice HRCT protocol showed good accuracy and sensitivity of 93% and 88%, respectively, compared with the standard whole-chest HRCT (64-slice or 128-slice) in 205 systemic sclerosis (SSc) patients [40]. However, in less developed countries, the availability of low radiation protocols and HRCT facilities may be limited, and the high cost may restrict its use for screening and monitoring purposes. Also, as RA is a very prevalent disease, it may not be appropriate to screen all RA patients for ILD with HRCT.

Serial PFTs have been used to monitor patients with CTD-ILD and are frequently used in clinical practice as well as in clinical trials [41, 42]. Forced vital capacity (FVC) and diffusing capacity for carbon monoxide (DLco) are important pulmonary function parameters for assessing lung physiology, including in RA-ILD [43]. The predicted FVC% and DLco% value could help guide management strategies and predicts mortality. However, its role in screening for early asymptomatic ILD is controversial. Recently, a study by Suliman et al. demonstrated a high risk of missing significant SSc-related ILD when relying solely on PFTs [44]. Among 102 SSc patients, 64 (63%) showed significant ILD on HRCT, while only 27 (26%) had an FVC < 80% predicted, and 54 (53%) had a decrease in the results of at least 1 PFT. Forty (62.5%) of 64 patients with significant ILD on HRCT had normal FVC, translating into a high false-negative rate [44]. These results were confirmed by Hoffmann-Vold et al. in a prospective cohort study including 305 SSc patients [45]. There exists no data in this regard for RA-ILD, but it is highly probable that comparable results hold true for RA. Therefore, more sensitive and repeatable methods for screening RA patients for ILD are highly on demand.

### LUS for CTD-ILD

In the past two decades, LUS has evolved as a promising tool in the assessment of pulmonary parenchymal diseases [46]. The inherent characteristics of ultrasound, including that it is non-ionizing, non-invasive, at low cost, repeatable, and easily accessible, make LUS a possible initial screening tool. LUS has been proposed to assess the extent and severity of ILD by detecting and quantifying the number of lung comet tail signs (B-lines) that originate from thickened septa [47]. B-lines are defined as discrete laser-like vertical hyperechoic reverberation artifacts that arise from the pleura, extend to the bottom of the screen without fading, and move synchronously with respiration [48]. B-lines are visible when the lung parenchymal air content is partially decreased and/or the interstitial space is volumetrically expanded, such as in pulmonary edema and/or ILD [49–51]. Despite LUS with the assessment of B-lines is appealingly simple to use, to learn, and to teach, sufficient theoretical and practical skills and training are prerequisites [52]. International evidence-based consensus recommendations for point-of-care lung ultrasound from Volpicelli et al. are helpful in guiding the implementation, development, and standardization of LUS across a variety of clinical settings [48].

Currently, different LUS scoring systems (total lung scanning sites range from 10 to 72) to assess and quantify the severity of CTD-ILD have been developed [53]. More scanning sites will undoubtedly be more accurate; however, this is at the cost of requiring more time to do the scanning. Although the full validation of LUS in CTD-ILD has not been completed, the data on early screening and diagnosis of ILD are encouraging [14, 54].

Recently, LUS and HRCT were used to screen early ILD change in 64 asymptomatic RA patients. LUS examination revealed that 18 patients (28%) had sonographic ILD (Table 1). In 16 (89%) LUS-positive patients, HRCT scans confirmed the ILD diagnosis [55]. More data are derived from ILD in SSc patients. Barskova et al. first used LUS for the screening of ILD in very early SSc patients. The concordance rate between B-lines and HRCT for the assessment of ILD was 0.83, and B-line numbers were significantly different in patients with and without HRCT-ILD (57 ± 53 vs 9 ± 9, *p* < 0.05) and with and without ground glass opacity (GGO) on HRCT (63 ± 47 vs 33 ± 40, *p* < 0.05). These data suggest that LUS has high sensitivity to identify ILD in SSc [56]. A prospective study in 133 SSc patients showed that LUS findings correlated with HRCT (*p* = 0.001) and these preliminary data revealed high sensitivity and specificity of LUS to detect ILD [57]. Çakir et al. investigated the ability of LUS to assess ILD severity in 48 SSc patients, showing a good correlation between B-lines, HRCT (*r* = 0.89, *p* = 0.0001), and the Medsger disease scale (*r* = 0.55, *p* = 0.0001), and a negative correlation with DLco (*r* = −0.56, *p* = 0.0001) and FVC (*r* = −0.46, *p* = 0.001). The diagnostic accuracy of LUS was comparable to HRCT [58]. In another study, LUS was performed in 104 patients undergoing HRCT for suspected ILD. According to HRCT, ILD was present in 50 patients. The false-negative and false-positive numbers of LUS were 4 (8%) and 11 (22%), respectively, compared to HRCT as the gold standard. The study concluded that LUS could be a sensitive tool for ILD detection, although the data point to the need for follow-up when LUS is abnormal [37]. In addition, LUS could detect alveolar-interstitial involvement (an early sign of ILD) in 31 patients with CTD [59]. These promising data support LUS as a screening tool for the diagnosis of early ILD in RA (Table 1).

**Table 1:** Overview of included studies

Author	Number of patients	Aim of study	Comparison with other diagnostic modality	Feasibility	Cutoff value of B-line number	Sensitivity (%)	Specificity (%)	NPV (%)	PPV (%)	AUC (%)
Moazedi-Fuerst et al. [55]	64 RA patients	To screen subclinical RA-ILD	HRCT	N/A	N/A	97.1	97.3	98.6	94.3	N/A
Barskova et al. [56]	58 SSc patients, including 32 VEDOSS	To screen early SSc-ILD	HRCT	100%	> 5 ≥20	100 83	55 96	100 N/A	78 N/A	94 N/A
Gutiérrez et al. [57]	133 SSc patients	To detect and predict asymptomatic SSc-ILD	HRCT	N/A	N/A	91.2%	88.6%	N/A	N/A	N/A
Çakir et al. [58]	48 SSc patients	To evaluate the severity of SSc-ILD	HRCT	N/A	≥6 > 24	100 79.3	84.2 94.7	100 N/A	90.6 N/A	93.7 94.8
Vizioli et al. [37]	104 suspected ILD patients	To evaluate the accuracy of LUS detection of ILD	HRCT	100%	> 5 > 10	92 92	53 66	87 90	64 71	90 N/A
Aghdashi et al. [59]	31 suspected rheumatoid lung involvement patients	To investigate the utility of LUS	HRCT	N/A	> 5	73.5	88.2	51.7	95.1	N/A

*AUC*, area under the curve; *HRCT*, high-resolution computed tomography; *ILD*, interstitial lung disease; *LUS*, lung ultrasound; *N/A*, not applicable; *NPV*, negative predictive value; *PPV*, positive predictive value; *RA-ILD*, rheumatoid arthritis–associated interstitial lung disease; *SSc*, systemic sclerosis; *SSc-ILD*, systemic sclerosis–associated interstitial lung disease; *VEDOSS*, very early diagnosis of systemic sclerosis

Recently, Tardella et al. explored the optimal cutoff values of numbers of B-line to predict the presence of significant ILD in 40 SSc patients [60]. An excellent correlation between the LUS B-line number and HRCT Warrick score was confirmed (Spearman rho 0.958, *p* = 0.0001). The receiver operating characteristic curve analysis revealed that the presence of 10 B-lines is the cutoff point with the greatest positive likelihood rate (12.52) for the presence of significant SSc-ILD (Warrick score ≥ 7). The value represents the best compromise between the best sensitivity (96.3%) and specificity (92.31%) by HRCT [60].

Gargani et al. preliminarily assessed the prognostic value of LUS B-lines to predict new development or worsening of pulmonary involvement in a total of 396 consecutive patients with SSc enrolled from three rheumatology departments. In the multi-variable analysis, the number of posterior B-lines ≥5 was associated with new development or worsening of ILD (hazard ratio, 3.378; 95% CI, 1.137–9.994; *p* = 0.028), performed better than anti-topoisomerase I antibody positivity [61].

These promising findings support LUS both as a screening and following tool for ILD in SSc and, hopefully, also other CTDs including RA.

### KL-6 for CTD-ILD

A biomarker may be defined as “any substance, structure, or process that can be measured in the body or its products and influences or predicts the incidence of outcome or disease” [62]. There is growing evidence that some circulating serological and alveolar biomarkers could reflect pathological processes, from early alveolar epithelial cell damage to advanced fibrosis. Among them, KL-6 has been extensively studied and it has emerged as a potentially sensitive surrogate marker of the presence of CTD-ILD and its severity [63–65]. KL-6 antigen is a mucin-like, high molecular weight glycoprotein expressed on the surface membrane of alveolar epithelial cells and bronchiolar epithelial cells, which increases following cellular injury and/or regeneration [66]. Its pathogenic role in pulmonary fibrosis is suggested by its pro-fibrotic, anti-apoptotic effects on fibroblasts [67].

Multiple studies indicated that serum and bronchoalveolar lavage fluid KL-6 levels were significantly correlated with HRCT findings and PFT variables in CTD-ILD [68, 69]. KL-6 concentrations were significantly higher in patients with ILD compared to those without ILD and showing a correlation to the ILD course [70, 71]. Furthermore, KL-6 had predictive value for the development and progression of ILD [72–75]. High KL-6 levels (≥ 640 U/mL) were independently associated with a UIP pattern (OR, 5.173; *p* = 0.05) in RA-ILD [76]. KL-6 levels could reflect early pulmonary epithelial cell injury and increased alveolar-capillary permeability [77]. Serum KL-6 levels were also associated with alveolitis in 66 SSc patients. The receiver operating characteristic curve analysis to evaluate the accuracy of KL-6 for the diagnosis of active alveolitis showed that 500 U/mL was the best cutoff value with a sensitivity of 78.8% and specificity of 90% (area under the curve (AUC) = 0.90) [78].

### LUS B-lines correlated with KL-6 in CTD-ILD

In a retrospective study, comprised of 60 confirmed CTD-ILD patients, including 11 patients with RA-ILD, circulating KL-6 levels correlated positively with the LUS B-line score (*r* = 0.54, *p* < 0.0001) [17]. The significant relationship was confirmed in 38 patients with IIM-ILD (*r* = 0.43, *p* < 0.01) [18]. In a recent case, B-lines and KL-6 were utilized to closely detect and follow a patient with anti-MDA-5-positive, clinically amyopathic dermatomyositis associated with rapidly progressive ILD [79]. Changes in B-line numbers and serum levels of KL-6 were consistent with the changes in HRCT findings and clinical presentation. Basing the treatment decision on B-line scores and KL-6 values, the patient was successfully rescued and avoided excessive radiation exposure. The case indicated that combining lung ultrasound and serum biomarkers might be a possibility for monitoring rapidly progressive ILD.

## A preliminary proposal of an algorithm for the use of LUS and KL-6 to screen and follow up early RA-ILD

**Fig. 1:**
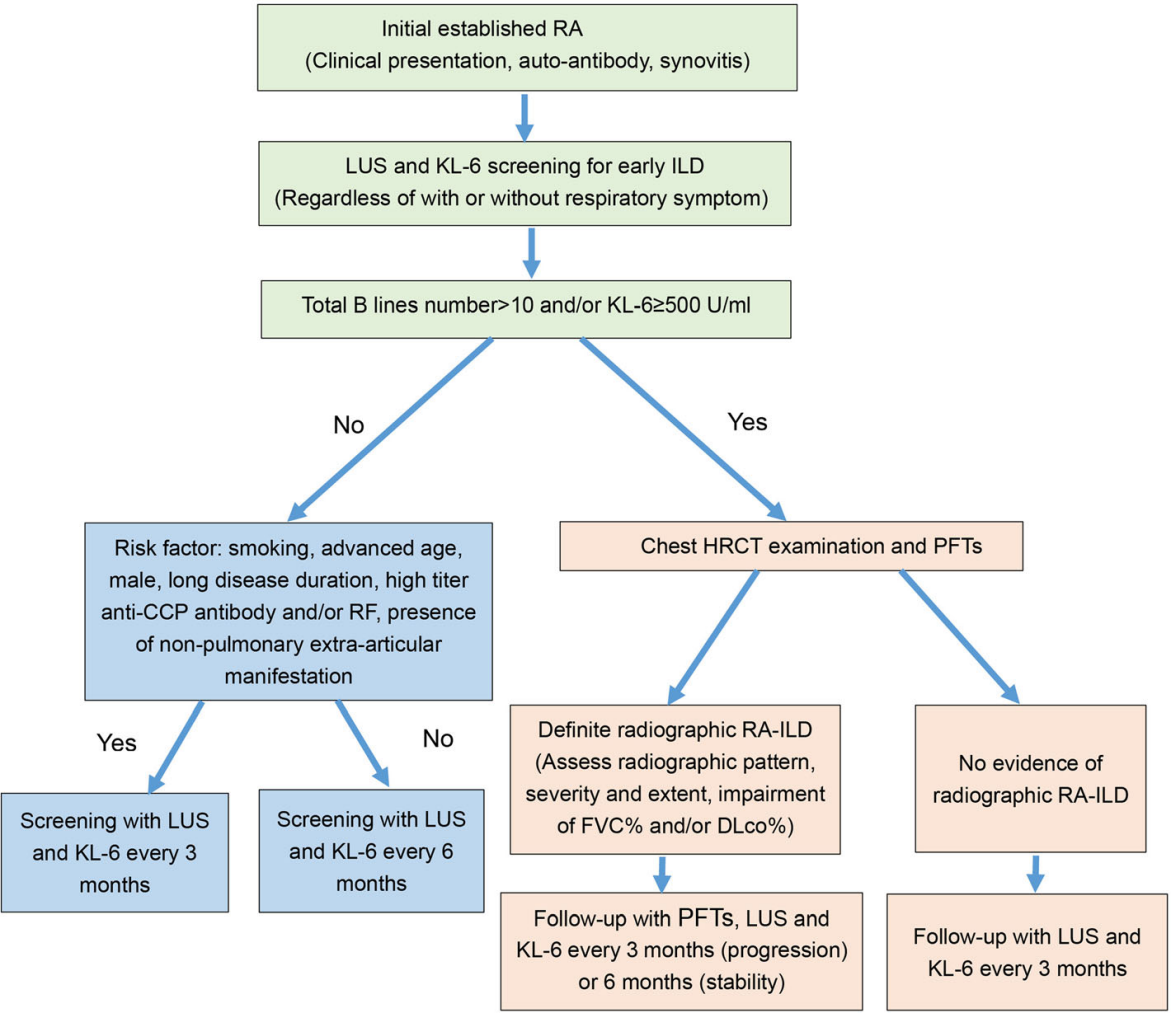
Preliminary algorithm for screening and follow-up of early RA-ILD. CCP, cyclic citrullinated peptide; DLco, diffusing capacity for carbon monoxide; FVC, forced vital capacity; HRCT, high-resolution computed tomography; ILD, interstitial lung disease; KL-6, Krebs von den Lungen-6 antigen; LUS, lung ultrasound; PFTs, pulmonary functional tests; RA, rheumatoid arthritis; RA-ILD, rheumatoid arthritis–associated interstitial lung disease; RF, rheumatoid factor

Based on the above literature and our clinical experience, we propose a preliminary algorithm for the screening and follow-up of early RA-ILD (Fig. 1).

1. Use LUS and KL-6 to screen all RA patients for the presence of ILD early in the disease course.
2. Patients without lung involvement (with total B-line number ≤ 10 and serum KL-6 levels < 500 U/mL) could be monitored every 3 to 6 months (depending on the presence of risk factors for RA-ILD) using LUS and KL-6.
3. If ILD is suspected from the first examinations (total B-line number > 10 and/or KL-6 ≥ 500 U/mL), patients should undergo a chest HRCT and PFTs to diagnose ILD and assess the extent of anatomic (HRCT of the chest) and physiologic (PFTs) involvement.
4. For patients with definite radiographic ILD, LUS, KL-6, PFTs, and clinical assessment could be performed to follow up patients every 3 to 6 months according to clinical behavior (progressive or stable).
5. If HRCT does not confirm ILD, patients could be screened by LUS and KL-6 every 3 months.

While we believe that adding LUS and KL-6 to PFTs and HRCT will be useful, we realize that this is a preliminary proposal and further study will be needed to validate and expand this proposal. For example, there needs to be agreement on the appropriate method(s) to measure KL-6 and it will need to be widely available. Likewise, LUS will need to be taught and standardized. Also, the definition of meaningful changes in LUS and KL-6 is still needed. Further, the most useful combination of LUS, KL-6, and PFTs for greatest sensitivity and accuracy to define stability and change will need careful study as it is expected that using all 3 variables in concert will be the best way forward. This preliminary approach may help in clinical practice while we wait for its validation. Herein, we present two cases as examples of applying this strategy to identify early ILD involvement in two anti-CCP antibody- and RF-positive juvenile arthritis patients.

## Case presentation

### Case 1

**Fig. 2:**
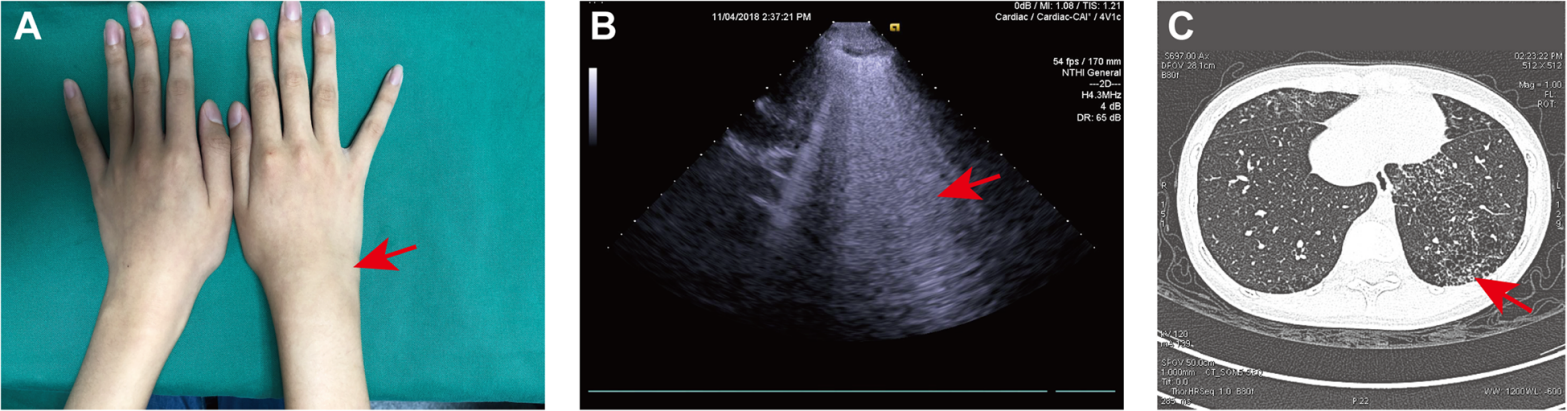
Case 1. **A** Physical examination showed right wrist swelling (arrow). **B** Lung ultrasound revealed multiple B-lines (arrow). **C** HRCT demonstrated diffuse ground glass opacity, reticular abnormalities, and traction bronchiectasis (arrow)

A 16-year-old non-smoking female patient complained of right wrist pain, swelling, and morning stiffness for 6 months. She denied symptoms of dry cough, shortness of breath, exertional dyspnea, and chest pain. Physical examination showed right wrist swelling and tenderness. Pulmonary auscultation was normal and no finger clubbing was evident. Laboratory results revealed high titer anti-CCP antibody (500 U/L, reference < 17 U/L) and RF (245 IU/mL, reference < 20 IU/mL) and increased erythrocyte sedimentation rate (ESR 30 mm/H, reference < 15 mm/H) and C-reactive protein (CRP 13 mg/L, reference < 8 mg/L). The disease activity score 28 (DAS28)-CRP was 2.8. Serum procalcitonin level was normal and the interferon gamma release assay (IGRA) test for tuberculosis was negative. Hand/wrist magnetic resolution imaging (MRI) showed synovitis and bone marrow edema. The LUS examination found a large number of B-lines in the whole lungs (total number 237) and serum KL-6 level was 500 U/L. Consequently, a chest HRCT was conducted and showed diffuse GGO, reticular abnormalities, and traction bronchiectasis corresponding to the NSIP pattern (Fig. 2). Moreover, PFTs showed a restrictive pattern with significant interstitial involvement (FVC 57.2%, forced expiratory volume in the 1st second [FEV1] 59.4%, DLco 56.4%, total lung capacity [TLC] 69.6%). The diagnosis of RA-ILD was made. No infection or other lung lesions were identified. Given that methotrexate and leflunomide may have lung toxicity and anti-TNF-α could exacerbate existing ILD [80], tofacitinib (5 mg twice daily) was chosen as a disease-modifying anti-rheumatic drug (according to recent observational studies showing that Janus Kinase inhibitors had efficacy on IIM-ILD) [81, 82]. She was additionally treated with low-dose prednisone (10 mg daily), celecoxib (200 mg daily), and carbocysteine (500 mg three times per day). After 3 months, joint symptoms improved (DAS28-CRP was 2.6). Follow-up with LUS examination (B-line number 240) and serum KL-6 level (480 U/L) indicated stable ILD.

### Case 2

**Fig. 3:**
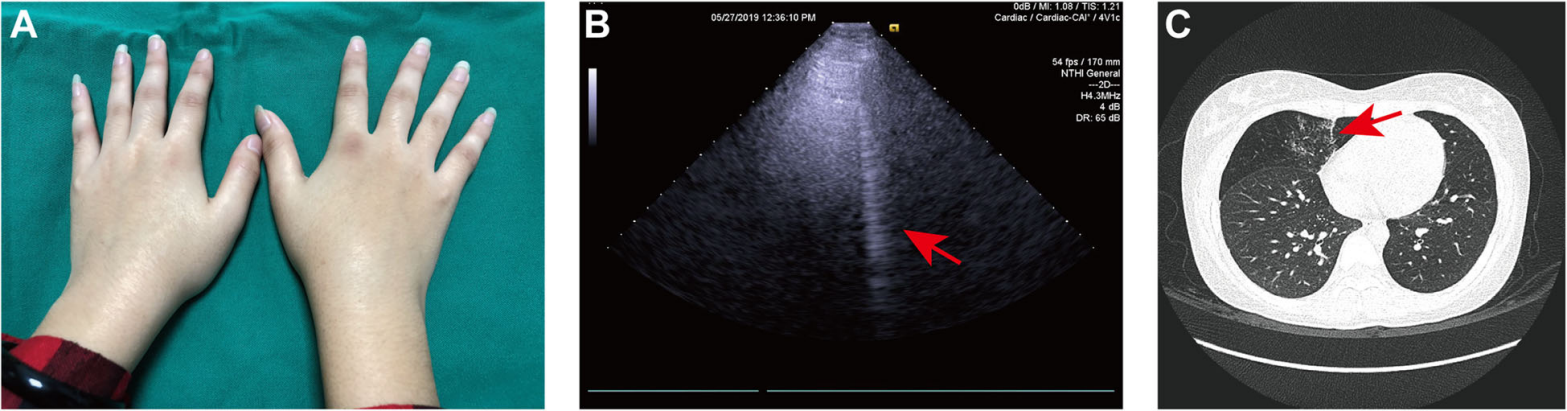
Case 2. **A** Physical examination showed polyarthritis of the hand joint. **B** Lung ultrasound revealed B-lines (arrow). **C** HRCT demonstrated ground glass opacity and interlobular septal thickening pattern located at the anterior segment of the right upper lobe (arrow)

A 15-year-old non-smoking female patient presented with symmetrical hand proximal interphalangeal (PIP) and metacarpophalangeal (MCP) joint swelling and pain (14 tender joints, and 12 swollen joints) for 3 months, and morning stiffness lasting longer than 60 min. She felt fatigue and did not present with cough or dyspnea. Laboratory results showed high titer anti-CCP antibody (500 U/L) and RF (930 IU/mL) and elevated ESR (72 mm/h) and CRP (27.6 mg/L). Anti-nuclear antibody, extractable nuclear antibody, and IGRA test were negative, and procalcitonin level was normal. Musculoskeletal ultrasound visualized synovitis and tenosynovitis. DAS28-CRP was 5.3. A diagnosis of juvenile RA was made, and the patient was screened with LUS examination that showed an increased number of B-lines (total number 63), while KL-6 levels were normal (151 U/L). Chest HRCT revealed GGO with an NSIP pattern. In the PFTs, only DLco% (62%) was reduced (Fig. 3). Rheumatology’ workup, laboratory, and imaging results confirmed asymptomatic RA-ILD. She received tofacitinib (5 mg twice daily), hydroxychloroquine (200 mg daily), low-dose prednisone (10 mg daily), celecoxib (200 mg daily), and carbocysteine (500 mg three times per day). After 3 months of therapy, her articular signs and symptoms improved. At LUS follow-up, the number of B-lines had decreased to 54, while PFT examination revealed DLco% was unchanged at 64%. HRCT was not repeated to avoid additional radiation.

The clinical features of the aforementioned two juvenile RA cases are young female, with short disease duration, without other risk factors such as smoking or presence of respiratory symptoms, except for the positive anti-CCP antibody. We used LUS and KL-6 as preliminary screening tools and identified the early ILD, which was confirmed by HRCT and PFTs.

## Considerations for a new strategy to screen and follow up RA-ILD

This algorithm seems to be easily applicable and readily available in most countries but needs to be tested and validated in a large number of patients. In the future, testing could be done by establishing a cohort of RA patients who undergo baseline examinations including auscultation, PFTs, HRCT, and assessment of respiratory symptoms and quality of life, as well as LUS and KL-6. Patients with diagnosed RA-ILD should be treated as clinically indicated. Patients without initial ILD diagnosis should undergo every 3–6 months LUS and KL-6 examination taking the patient’s risk profile into account. All patients would undergo annual testing with HRCT, PFTs, LUS, and KL-6. If predefined changes in HRCT, PFTs, or symptoms occur, treatment adjustment should be undertaken as clinically indicated. Follow-up should be conducted for 3 years. The clinical utility of LUS and KL-6 can thus be tested and validated in terms of its minimum clinically important change, positive predictive value, and negative predictive value relative to either predefined changes in HRCT and/or PFT or clinical need to treat.

## Conclusion

LUS and serum KL-6 are inexpensive, non-invasive, and radiation-free measures which may be used to screen RA patients for the presence of ILD. These biomarkers could be employed as preliminary measures, together with respiratory symptoms and followed by HRCT and PFT, in order to enable the early diagnosis of RA-ILD. Prospective testing and validation of this concept are necessary.

## Data Availability

Not applicable.

## Abbreviations

AUC: Area under the curve
CCP: Cyclic citrullinated peptide
CRP: C-reactive protein
CTD-ILD: Connective tissue disease–associated interstitial lung disease
DAS28: Disease activity score 28
DLco: Diffusing capacity of carbon monoxide
ESR: Erythrocyte sedimentation rate
FEV1: Forced expiratory volume in the first second
FVC: Forced vital capacity
GGO: Ground glass opacity
HRCT: High-resolution computed tomography
IGRA: Interferon gamma release assay
IIM-ILD: Idiopathic inflammatory myositis–related interstitial lung disease
ILD: Interstitial lung disease
KL-6: Krebs von den Lungen-6 antigen
LUS: Lung ultrasound
MCP: Metacarpophalangeal
MRI: Magnetic resolution imaging
NPV: Negative predictive value
NSIP: Non-specific interstitial pneumonia
PFTs: Pulmonary function tests
PIP: Proximal interphalangeal
PPV: Positive predictive value
RA: Rheumatoid arthritis
RA-ILD: Rheumatoid arthritis–associated interstitial lung disease
RF: Rheumatoid factor
SSc: Systemic sclerosis
TLC: Total lung capacity
TNF-α: Tumor necrosis factor-α
UIP: Usual interstitial pneumonia
